# Bilateral Primary Non-Hodgkin’s Lymphoma of the Lacrimal Sac: A Case Report

**DOI:** 10.7759/cureus.29114

**Published:** 2022-09-13

**Authors:** Mohamed Noor Arjamilah, Akmal Haliza Zamli, Evelyn Tai, Ismail Shatriah

**Affiliations:** 1 Opthalmology, Hospital Tengku Ampuan Afzan, Kuantan, MYS; 2 Ophthalmology and Visual Science, School of Medical Sciences, Universiti Sains Malaysia, Kubang Kerian, MYS; 3 Ophthalmology, Hospital Tengku Ampuan Afzan, Kuantan, MYS

**Keywords:** chemotherapy, endoscopic dacryocystorhinostomy, dacryocystitis, lacrimal sac, primary non-hodgkin's lymphoma

## Abstract

Primary non-Hodgkin’s lymphoma of the lacrimal sac is extremely rare, usually representing secondary involvement of systemic malignancy. We report a case of bilateral primary non-Hodgkin’s lymphoma of the lacrimal sac presenting with bilateral medial canthal swelling for one month which was preceded by a history of chronic bilateral epiphora and a recurrent history of dacryocystitis. The symptoms partially responded to systemic antibiotics for the past three years. Clinical examination revealed bilateral diffuse erythematous medial canthal swelling extending to the upper cheeks. CT of the orbits and paranasal sinuses demonstrated soft tissue masses involving bilateral lacrimal sacs and ducts. Endoscopic dacryocystorhinostomy (DCR) with excision biopsy of both lacrimal sac was performed and histopathologically confirmed the diagnosis of extranodal marginal zone B-cell lymphoma. She completed six cycles of chemotherapy. The symptoms subsided and radiologically showed a significant reduction of soft tissue mass at bilateral nasolacrimal sacs and ducts after completion of chemotherapy. Recurrent atypical presentation of dacryocystitis with suboptimal response to standard treatment should raise a suspicion of secondary cause. Histopathological examination is therefore crucial to avoid delays in diagnosis and treatment.

## Introduction

Dacryocystitis is an infection of the lacrimal sac which usually results from nasolacrimal duct obstruction. The most common cause is primary acquired nasolacrimal duct obstruction, with histologic findings of chronic inflammation and fibrosis leading to occlusion of the lacrimal drainage system, while secondary acquired nasolacrimal duct obstruction can result from neoplasm, systemic inflammatory disease, infection, or trauma. Epiphora, redness, and swelling of the medial canthus are the most common clinical manifestations [1].

Lymphoproliferative orbital diseases are responsible for 10%-15% of all ocular tumors as secondary causes [2]. It may originate in the periorbital and/or orbital region (primary lymphoma) or may appear in this region as a result of systemic spread (secondary lymphoma) [3]. Primary non-Hodgkin’s lymphoma of the lacrimal sac is rare. The vast majority of lymphomas involving the lacrimal sac are secondary [4]. The clinical manifestations can be misleading as it mimics primary acquired nasolacrimal duct obstruction [5-6].

Herein, we report a rare case of bilateral primary non-Hodgkin’s lymphoma of the lacrimal sac that manifested clinically as recurrent dacryocystitis.

## Case presentation

A 65-year-old Malay lady with underlying diabetes mellitus, hypertension, and hyperlipidemia presented with bilateral medial canthal swelling for one month. She gave a history of chronic bilateral epiphora with recurrent acute dacryocystitis, which partially responded to systemic antibiotics. Otherwise, there was no history of nasal congestion, epistaxis, or sinusitis. She underwent right endonasal dacryocystorhinostomy (DCR) surgery three years ago, but her symptoms persisted and recurred a year later.

**Figure 1:**
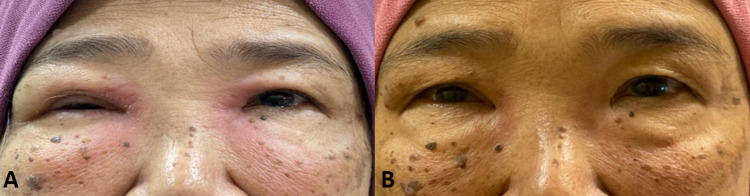
A. The clinical appearance of both medial canthal swellings before chemotherapy. B. Both medial canthal swellings subsided post chemotherapy.

Clinical examination revealed bilateral diffuse erythematous medial canthal swelling extending to the upper cheeks (Figure 1A). A positive dye disappearance test was observed bilaterally suggestive obstruction. Otherwise, the anterior and posterior segments of both eyes were normal. There was no lymphadenopathy, hepatosplenomegaly, or focal neurologic deficit to suggest systemic involvement. 

**Figure 2:**
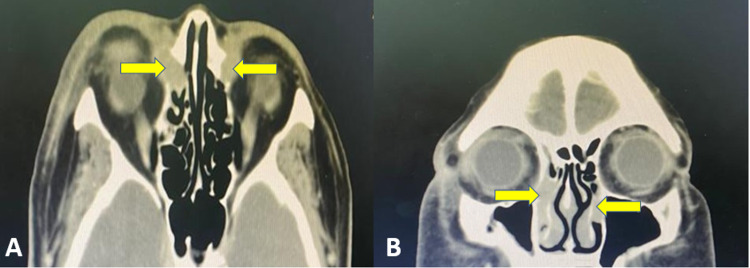
A) Axial and B) coronal view of CT scan showing a homogenous soft tissue mass in the lacrimal sac which is isodense with respect to extraocular muscle (yellow arrow).

CT of the orbits and paranasal sinus revealed bilateral soft tissue masses involving the bilateral lacrimal sac measuring approximately 15.85 mm (AP) x 20.99 mm (CC) x 11.78 mm (W) on the right side and 15.83 mm (AP) x 15.57 mm (CC) x 9.27 mm (W) on the left side. Both masses extend into the nasolacrimal ducts till the mid-portion of the inferior turbinates below the valve of Taillefer (Figure 2A-B).

The patient then underwent bilateral endoscopic DCR (revision of the right) with excision biopsy. Intra-operative findings revealed fragile, lobulated granulation tissue in both lacrimal sacs.

**Figure 3:**
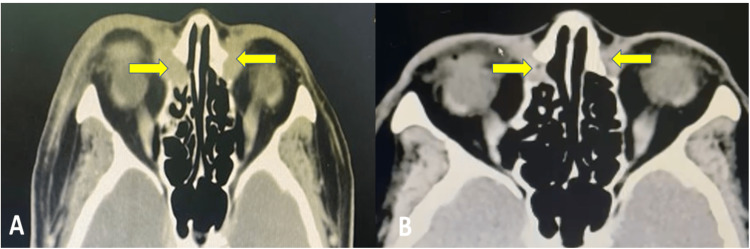
A) Pre-chemotherapy and B) Post-chemotherapy of CT scan (axial view) showing reduction in the size of homogenous soft tissue mass in lacrimal sac.

The histopathological examination showed features of low-grade B cell lymphoma suggestive of extranodal marginal zone lymphoma. CT of the thorax, abdomen, and pelvis for staging was performed and showed no evidence of systemic involvement. She was treated with six cycles of chemotherapy (chlorambucil with prednisolone). After six cycles of chemotherapy, the bilateral epiphora and medial canthal swelling resolved, with repeated CT of the orbits and paranasal sinus showed a reduction in the size of the bilateral nasolacrimal sac and duct soft tissue masses (Figure 3A-B). 

## Discussion

Primary lacrimal sac tumor is an extremely rare disease. It can be classified into epithelial and non-epithelial in origin, which constituted 73% and 27%, respectively [7]. Non-epithelial tumors are further subdivided into mesenchymal tumors, lymphomas, malignant melanoma, and neural tumors. Among all these tumors, lymphoma comprises only 8%, which is extremely low [7] and often arises secondary to systemic lymphoproliferative disorders [8-9].

The lacrimal sac is a mucosal component of the lacrimal drainage system that contains well-described aggregates of lymphoid tissue known as lacrimal drainage-associated lymphoid tissue (LDALT), which is synonymous with mucosa-associated lymphoid tissue (MALT) and contributes innately to the mucosal immune response independent of the systemic immune system. Furthermore, the highest topographical concentration of LDALT within the lacrimal drainage system is in the lacrimal sac and duct. Although uncommon, it is possible for lymphoma to originate in the lacrimal sac [10].

Painless swelling over the medial canthal region accompanied by epiphora may suggest a lacrimal sac tumor. However, epiphora precedes tumor palpation for several months in lacrimal sac MALT lymphoma [11-12]. Diagnosis of a lacrimal sac tumor is difficult without palpation, as epiphora is a non-specific symptom that is also present in acquired nasolacrimal duct obstruction [11]. Sjö et al. reported that the most common symptoms were epiphora (85%), followed by medial canthal swelling (79%) and dacryocystitis (21%) [3]. Schefler et al. reported additional symptoms of diplopia, and nasal symptoms such as sinusitis, nasal obstruction, or epistaxis depend on the spread of the disease [12]. In our case, the patient presented with a recurring atypical presentation that resembled chronic dacyrocystitis without a palpable mass or sinister nasal symptoms, as previously reported in studies. Hence, the diagnosis of primary nasolacrimal duct obstruction was made initially, causing a delay in diagnosis and treatment. Chronic inflammation in mild lacrimal sac MALT lymphoma can cause secondary obstruction of the nasolacrimal duct, which has a clinical profile similar to primary acquired nasolacrimal duct obstruction resulting in diagnostic delays, as in our case [13].

In the last 30 years, less than 50 cases of primary lacrimal sac lymphoma have been reported [14]. However, most studies of primary lacrimal sac lymphoma have been case reports, with no imaging features included. This lesion is difficult to diagnose on imaging and is often misdiagnosed as carcinoma, pseudotumors, or inflammatory lesions. Guo et al. reported tumors of the lacrimal sac showed homogeneous and isointense patterns on T1WI and T2WI when compared to adjacent extraocular muscles, which is the key to the diagnosis. There is mild to moderate enhancement and a plateau pattern on dynamic contrast-enhanced (DCE) MRI, while CT showed remodeling of the lacrimal duct with bone compression. CT is sensitive for the evaluation of bony erosion, while MRI is valuable for evaluating the location, form, margin, and invasion of adjacent structures [15]. In our case, the CT of the orbits was suggestive of a benign lesion in the lacrimal sacs and ducts and radiologically not suggestive of lymphoma. MRI would be more appropriate in this situation as it is superior to CT scanning in assessing soft tissue. However, the patient was not arranged for MRI as an excision biopsy was planned for a definitive diagnosis.

The role of routine biopsy during DCR remains controversial. The initial advocates for routine biopsy were Linberg and McCormick, who revealed that 1 in 16 routine DCR biopsies (7.5%) was unexpected leukemia, which is still low in percentage [16]. Bernardini et al. concluded that only patients with a known pre-existing systemic disease or an abnormal lacrimal sac intraoperatively had a "positive" histopathologic diagnosis [17]. As in our case, the patient’s histopathology confirmed extranodal marginal zone lymphoma despite not being known to have lymphoproliferative diseases. Thus, in the context of any suspicious clinical sign, a biopsy should be performed [13].

The management of primary lacrimal sac lymphoma is not well established due to the rarity of the disease. The treatment modalities consist of surgery, radiotherapy, chemotherapy, or a combination. Most of the primary lacrimal sac lymphoma cases in the literature were treated with radiotherapy with or without chemotherapy and surgery [3, 12]. However, in our case, the patient was successfully treated with surgery in addition to chemotherapy, and she achieved remission after four months of chemotherapy completion (Figure 1B).

## Conclusions

Nasolacrimal duct obstruction with atypical presentation and suboptimal response to standard treatment should raise suspicion of a more malignant cause. Therefore, histopathological examination is crucial to aid diagnosis in these cases. 

## References

[1] Noor SIN, Ramiza RR, Adil H, Hasnan J (2017). Primary non hodgkin's lymphoma of lacrimal sac presented as recurrent acute dacryocystitis. J Acute Dis.

[2] Knowles DM 2nd, Jakobiec FA (1982). Ocular adnexal lymphoid neoplasms: clinical, histopathologic, electron microscopic, and immunologic characteristics. Hum Pathol.

[3] Sjö LD, Ralfkiaer E, Juhl BR (2006). Primary lymphoma of the lacrimal sac: an EORTC ophthalmic oncology task force study. Br J Ophthalmol.

[4] Montalban A, Liétin B, Louvrier C, Russier M, Kemeny JL, Mom T, Gilain L (2010). Malignant lacrimal sac tumors. Eur Ann Otorhinolaryngol Head Neck Dis.

[5] Chai CK, Tang IP, Tan TY (2013). Primary lacrimal sac lymphoma with recurrence. Med J Malaysia.

[6] Alkatan HM, Alaraj AM, Al-Ayoubi A (2012). Diffuse large B-cell lymphoma of the orbit: a tertiary eye care center experience in Saudi Arabia. Saudi J Ophthalmol.

[7] Parmar DN, Rose GE (2003). Management of lacrimal sac tumours. Eye.

[8] Ludwig MH, Anselm GMJ, Friedrich EK, Leonard MH (2010). Tumors of the lacrimal drainage system. Orbit.

[9] Pelloski CE, Wilder RB, Ha CS, Hess MA, Cabanillas FF, Cox JD (2001). Clinical stage IEA-IIEA orbital lymphomas: outcomes in the era of modern staging and treatment. Radiother Oncol.

[10] Knop E, Knop N (2001). Lacrimal drainage-associated lymphoid tissue (LDALT): a part of the human mucosal immune system. Investig Ophthalmol Vis Sci.

[11] Jones IS (1956). Tumors of the lacrimal sac. Am J Ophthalmol.

[12] Schefler AC, Shields CL, Shields JA, Demirci H, Maus M, Eagle RC Jr (2003). Lacrimal sac lymphoma in a child. Arch Ophthalmol.

[13] Abdelkhalek R, Ahmimech J, Mouzariae Y (2015). [Case of a bilateral MALT lymphoma of the lacrimal sac treated only medically]. J Fr Ophtalmol.

[14] Francis IC, Wilcsek G (2006). Expect the unexpected. Br J Ophthalmol.

[15] Guo P, Yan F, Tian C, Zhao P, Wang Z, Xian J (2014). Imaging and histopathological findings of lacrimal sac lymphoma. Chin Med J (Engl.

[16] Linberg JV, McCormick SA (1986). Primary acquired nasolacrimal duct obstruction. A clinicopathologic report and biopsy technique. Ophthalmology.

[17] Bernardini FP, Moin M, Kersten RC, Reeves D, Kulwin DR (2002). Routine histopathologic evaluation of the lacrimal sac during dacryocystorhinostomy: how useful is it?. Ophthalmology.

